# Neonatal Thyrotoxicosis in Infants of Mothers with Graves’ Disease Treated for Radioiodine-Induced Hypothyroidism: A Literature Review

**DOI:** 10.3390/children11080968

**Published:** 2024-08-11

**Authors:** Lucia Jankovski, Štefan Grosek, Mojca Tanšek Žerjav, Marijana Vidmar Šimic, Katja Zaletel

**Affiliations:** 1 Faculty of Medicine, University of Ljubljana, 1000 Ljubljana, Slovenia; lucia.jankovski@gmail.com; 2 Neonatology Section, Department of Perinatology, Division of Gynaecology and Obstetrics, University Medical Centre Ljubljana, 1000 Ljubljana, Slovenia; stefan.grosek@kclj.si; 3 Department of Medical Ethics, Faculty of Medicine, University of Ljubljana, 1000 Ljubljana, Slovenia; 4 Department of Pediatric Endocrinology, Diabetes and Metabolic Diseases, Division of Pediatrics, University Medical Centre Ljubljana, 1000 Ljubljana, Slovenia; mojca.zerjav-tansek@mf.uni-lj.si; 5 Department of Perinatology, Division of Obstetrics and Gynecology, University Medical Centre Ljubljana, 1000 Ljubljana, Slovenia; marijana.vidmarsimic@kclj.si; 6 Division of Nuclear Medicine, University Medical Centre Ljubljana, 1000 Ljubljana, Slovenia; 7 Department of Internal Medicine, Faculty of Medicine, University of Ljubljana, 1000 Ljubljana, Slovenia

**Keywords:** fetal thyrotoxicosis, neonatal thyrotoxicosis, Graves’ disease, stimulating antibodies against thyrotropin receptor, radioiodine treatment

## Abstract

Fetal and neonatal thyrotoxicosis occurs in up to 5% of pregnancies in mothers with Graves’ disease (GD). This condition is caused by stimulating antibodies against the thyrotropin receptor (TRAbs) that cross the placenta and may stimulate the fetal thyroid, typically in the second half of pregnancy. GD is often treated with radioiodine, resulting in hypothyroidism in most patients, but TRAbs can persist for several years. Even if a pregnant mother is hypothyroid after radioiodine therapy or surgery, her TRAbs can still, although rarely, induce fetal hyperthyroidism. In this review, we first present two cases of neonatal hyperthyroidism in mothers with GD who became hypothyroid after prior radioiodine therapy, identified through a 10-year analysis of the National Perinatal System in Slovenia. Based on these cases, we provide an overview of existing data on this rare clinical condition in neonates. We also discuss the underlying mechanisms and clinical outcomes based on currently available data. In conclusion, our review highlights the importance of careful monitoring during pregnancy in all women with GD, even in those well managed after radioiodine therapy or surgery.

## 1. Introduction

During pregnancy, hormonal and metabolic changes profoundly affect maternal thyroid function and thyroid autoimmunity. Early in gestation, human chorionic gonadotropin (hCG) stimulates thyrotropin (TSH) receptors, while elevated estrogen levels increase thyroxine-binding globulin and total thyroid hormone levels [1]. Maternal thyroid hormones, particularly free thyroxine (fT4), are crucial for fetal development in the first trimester [2]. Fetal thyroid development begins around the 7th week of gestation, with hormone production starting by the 10th–12th week and significantly increasing after the 18th–20th week. By the 25th week, the fetal thyroid is fully functional [3].

Hyperthyroidism may occur in up to 3% of pregnancies. The most common cause is gestational hyperthyroidism, a transient physiological condition induced by hCG during the first trimester that peaks around week 10 [1,4]. Hyperthyroidism due to pathological causes in pregnant women is rare, with an estimated prevalence of up to 0.4%, and Graves’ disease (GD) accounts for up to 90% of all cases [5,6]. Early diagnosis is crucial, as it can impact both the mother and fetus [7]. Overt hyperthyroidism may lead to complications, such as miscarriage, pre-eclampsia, premature labor, congestive heart failure in the mother and fetus, thyroid storm in the mother, intrauterine growth restriction, low birth weight, and fetal and neonatal thyrotoxicosis [8].

GD is an autoimmune condition characterized by stimulating antibodies, primarily belonging to the IgG2 class, against the TSH receptor (TRAbs) [9]. These antibodies induce thyroid hormone synthesis by binding to and activating TSH receptors on thyrocytes [10]. Newly diagnosed Graves’ hyperthyroidism is initially treated with antithyroid drugs to decrease thyroid hormone synthesis. If there is recurrence or unfavorable disease progression, treatment options include reducing the amount of thyroid tissue with radioiodine or, in rare cases, thyroidectomy. In such patients, the destruction or removal of thyroid tissue usually necessitates lifelong levothyroxine (L-T4) replacement to maintain euthyroidism, which can obscure the underlying diagnosis [11,12]. Although these patients may have elevated TRAbs for many years, the antibodies no longer pose a risk to them. However, during pregnancy, the antibodies may still present a risk and have harmful effects on the fetus and newborn [12].

TRAbs typically become problematic in the second half of pregnancy when their passage across the placental barrier increases [13] and the fetal thyroid matures enough for its TSH receptors to respond to them [3,6]. Fortunately, pregnancy-induced immunosuppression often leads to a significant decrease in maternal TRAb levels after the 20th week, sometimes reducing them to undetectable levels [6]. However, fetal and neonatal thyrotoxicosis can occur in up to 5% of pregnancies in mothers with GD, with a significant risk associated with a 3–5-fold increase in stimulating TRAb levels [14]. It is important to note that TRAbs can be present in women with active GD, as well as in situations where GD may not initially be suspected, such as women diagnosed with GD before pregnancy or those who have undergone radical treatments like radioiodine or surgery [15].

Given the rarity of fetal/neonatal thyrotoxicosis in mothers with GD and its serious health risks, the objective of our review was to provide an overview of existing data on this rare clinical condition in neonates based on nationwide case findings over a 10-year period. Additionally, we aimed to emphasize the importance of thorough patient history taking regarding current and prior treatment for GD, highlighting the necessity for frequent monitoring of maternal thyroid function and TRAb levels, as well as close fetal monitoring.

## 2. Methods

We initially retrospectively analyzed data from the National Perinatal Information System to identify cases of neonatal thyrotoxicosis in Slovenia, where the reported annual incidence of GD is approximately 300 new cases per million inhabitants, with affected individuals averaging 45 years of age [16]. Over a 10-year period from 1 January 2014 to 5 October 2023, during which Slovenia recorded between 17,627 and 21,165 live births annually, we identified three cases of neonatal thyrotoxicosis, all delivered at the University Medical Centre Ljubljana. All cases were treated between 1 May 2022 and 1 July 2023. Informed consent was obtained from two sets of parents, and approval of the Medical Ethics Committee of the Republic of Slovenia was granted. The parents of the third child did not consent to inclusion in the research; therefore, only two cases were evaluated.

From medical records, maternal history concerning GD was examined, including results of TRAb concentrations during pregnancy and delivery. In neonates, demographic, clinical, and biochemical characteristics were obtained as well as the data on treatment approach and clinical outcome. All results of TSH, fT4, free triiodothyronine (fT3), and TRAb were performed with the same laboratory platforms (Abbot Alinity, Chicago, IL, USA, for TRAb determination and Siemens Atellica, Erlangen, Germany, for all other measurements) using chemiluminescent methods.

**Figure 1:**
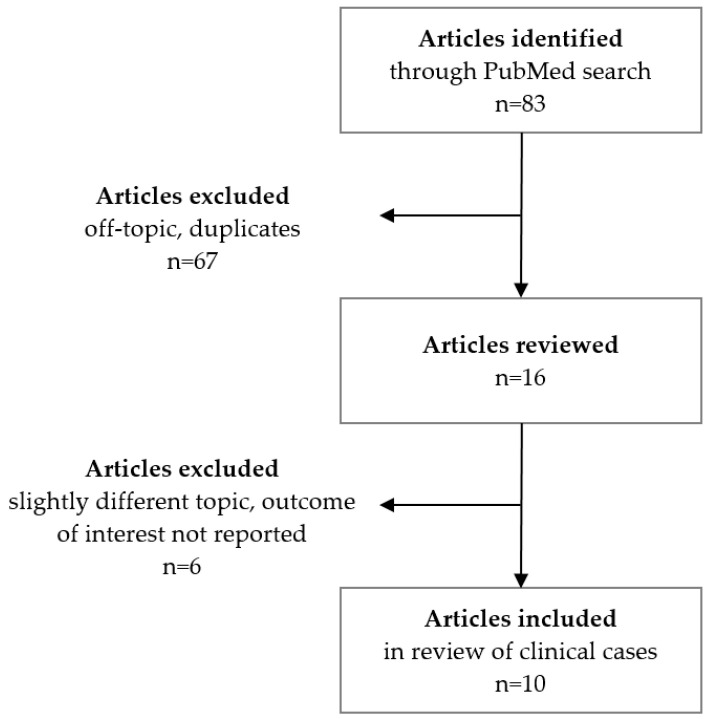
Data selection flowchart.

Furthermore, to identify relevant previous reports on fetal/neonatal thyrotoxicosis in mothers with GD who became hypothyroid after prior ablative therapy, we conducted a comprehensive search of the PubMed database. We selected pertinent case reports published from January 2000 to May 2024. The search utilized various combinations of the following terms: Graves’ disease; fetal; neonatal; offspring; hyperthyroidism; thyrotoxicosis; fetal goiter; hydrops; TSH receptor stimulating antibodies; TRAb; maternal hypothyroidism; radioiodine; thyroidectomy; and case. Articles that did not align with these topics were excluded from the review (Figure 1).

## 3. Case Presentations

**Figure 2:**
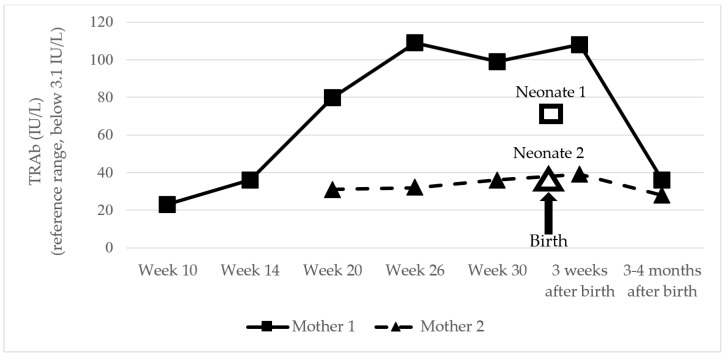
Stimulating antibodies against thyrotropin receptor (TRAb) in mothers (during pregnancy and after delivery) and neonates (at birth).

In our two cases, both mothers had been treated for hypothyroidism following radical treatment for GD over 10 years ago; both underwent radioiodine therapy and one additionally received a thyroidectomy (Table 1). During pregnancy, mother 1 was closely monitored by a thyroid specialist starting at week 10, while mother 2 began monitoring at week 20 when her pregnancy was confirmed. By adjusting L-T4 replacement therapy, euthyroidism was maintained in both mothers throughout pregnancy. As illustrated in Figure 2, TRAb levels in both mothers increased during pregnancy, continued until 3 weeks postpartum, and then sharply declined 3–4 months after delivery.

Both neonates were male and exhibited symptoms of hyperthyroidism from birth. Their clinical characteristics are outlined in Table 1. Both were born prematurely, with meconium-stained amniotic fluid. They had low birth weight and were small for their gestational age. The one- and five-minute Apgar scores for both were 9, and the arterial and venous umbilical cord pH levels were appropriate. They were transferred to the neonatal intensive care unit immediately after birth. Both exhibited tremors. Additionally, neonate 1 showed sinus tachycardia, irritability, and exophthalmos. A head ultrasound on day 2 revealed grade 2 intraventricular hemorrhage in neonate 1, while mild lateral ventricular asymmetry was observed in neonate 2.

In neonate 1, the TRAb concentration measured on day 6 was significantly elevated, although it was 30% lower than the mother’s level at 30 weeks of pregnancy. In neonate 2, the TRAb concentration at birth was similar to that of the mother at 30 weeks of pregnancy (Figure 2).

As presented in Table 1, neonate 1, who exhibited severe signs of hyperthyroidism, also showed more severe laboratory indicators of hyperthyroidism and higher TRAb concentrations. However, within 13 days (from day 6 to day 19 postpartum), the TRAb concentration decreased by 58%. In neonate 2, the TRAb concentration decreased by 44% within 10 days postpartum (from birth to day 10 postpartum).

Both neonates were prescribed methimazole, and at a dose of 0.4–0.5 mg/kg/day, free thyroid hormones significantly decreased within a few days. Additionally, neonate 1 received propranolol for treatment of tachycardia, as well as phenobarbital and chloral hydrate for irritability. Supplemental oxygen was given through the nasal cannula. Both neonates received enteral nutrition throughout their stay in the neonatal intensive care unit. Their symptoms significantly improved after the first two weeks of treatment, and they were discharged 2–3 weeks after birth.

## 4. Discussion with a Literature Review

Neonatal thyrotoxicosis in a mother being treated for hypothyroidism is exceedingly rare, with only a few reported cases to date. Over a 10-year period, we identified only three cases of neonatal thyrotoxicosis at the national level, with the mothers of two consenting cases having been treated for hypothyroidism following ablative thyroid therapy. This likely reflects both the rarity of the condition and the possibility that mild cases went unrecognized. This condition is caused by stimulating TRAbs present in the serum of mothers who were previously treated with radioiodine or surgery, as seen in our neonates [17,18]. Additionally, it can occur in mothers with Hashimoto’s thyroiditis who are treated for hypothyroidism and subsequently develop previously unrecognized TRAbs during pregnancy [19,20].

### 4.1. Review of Clinical Cases

Following a comprehensive search of the PubMed database (Figure 1), we identified 10 cases of fetal/neonatal thyrotoxicosis caused by TRAbs in mothers who were treated for hypothyroidism after thyroid ablation [21,22,23,24,25,26,27,28,29,30].

In hypothyroid mothers treated with radioiodine for GD prior to pregnancy, Garcia et al. reported two cases of neonatal thyrotoxicosis. The neonates, born at 36 and 35 weeks, were diagnosed in their third week due to the lack of TRAb monitoring during pregnancy [21]. Akingre et al. later described a neonate born at 33 weeks’ gestation who was diagnosed with TRAb-induced hyperthyroidism 3 weeks after birth. It was later discovered that the mother had a history of radioiodine treatment for GD, which had not been documented in her medical records [22]. Furthermore, Hong et al. reported two neonates born at 36 weeks, both closely monitored and treated with methimazole in the second half of pregnancy due to their mothers’ pregnancy history. Specifically, one mother had previously delivered a preterm neonate diagnosed with TRAb-induced hyperthyroidism at 1 month, while the other neonate experienced intrauterine death at 26 weeks due to fetal tachycardia, an enlarged heart, and oligohydramnios [23]. Recently, a case of neonatal thyrotoxicosis with serious complications was reported by Zhu et al. in a neonate diagnosed 15 days after birth, whose mother had undergone prior radioiodine treatment and lacked pregnancy monitoring [24]. However, van Hulsteijn et al. reported two hyperthyroid neonates in two separate pregnancies of the same mother who were closely monitored and received timely treatment without experiencing severe complications, highlighting the critical importance of appropriate management in such cases [25].

Although thyroidectomy is a significantly less frequently used method of radical GD treatment compared to radioiodine therapy [31], a few cases of neonatal hyperthyroidism following such maternal treatment have been reported. Bohîlțea et al. described a hyperthyroid neonate born at 35 weeks of gestation after close monitoring and methimazole treatment. However, after previous pregnancy, a newborn delivered at 31 weeks died 3 weeks after birth due to unrecognized and untreated hyperthyroidism [26]. Earlier, Kazakou et al. reported a neonatal death on day 5 despite treatment with methimazole following a fetal diagnosis of intrauterine growth retardation, oligohydramnios, worsening hydrops, and goiter at 29 weeks, leading to premature delivery at 30 weeks [27]. Recently, Fennell et al. described a case of fetal tachycardia and an enlarged right-sided heart identified during a routine prenatal ultrasound at 21 weeks of gestation, successfully treated with carbimazole during pregnancy, followed by delivery at 34 weeks and subsequent treatment with carbimazole combined with potassium iodide drops [28]. A similar case of hyperthyroidism was reported by Reineke et al., which was identified at 28 weeks, and the neonate, who was delivered at 36 weeks, was treated with propylthiouracil [29]. In an earlier report, Matsumoto et al. described two siblings in separate pregnancies with persistent tachycardia and cardiac failure, effectively treated from 23 and 22 weeks of gestation with propylthiouracil and potassium iodide, and with methimazole after birth at 36 and 35 weeks, respectively [30]. As in our cases, the reported maternal age in all cases was over 30 years.

### 4.2. Clinical Presentation of Fetal Thyrotoxicosis

Even though antibodies are transferred to the fetus early, the clinical signs of fetal hyperthyroidism typically appear between the 26th and 28th weeks, or, in rare severe cases, even earlier [6]. It can present with tachycardia, cardiac arrest, accelerated bone age, microcephaly, and craniosynostosis [32]. In our case, we also observed intraventricular hemorrhage. Also, the appearance of the femoral epiphysis before the 31st week of gestation, rather than the typical 32nd week, indicates advanced bone age, often due to hyperthyroidism [33]. If untreated, fetal thyroid stimulation in the second half of pregnancy can lead to intrauterine growth restriction, prematurity, hydrops, fetal death, and spontaneous abortion [32]. The mortality rate, which can reach up to 25%, is primarily attributed to congestive heart failure, intrauterine growth restriction, and premature delivery [26].

Monitoring the fetal thyroid gland plays a crucial role in mothers with GD, as studies have shown that fetal thyroid ultrasound at 32 weeks has a sensitivity of 92% and a specificity of 100% for diagnosing clinically relevant fetal thyroid pathology [34]. This observation aligns with several case studies of hypothyroid GD mothers with prior ablative therapy, which have reported goiter on fetal ultrasound evaluations [21,23,25,27,28,29,30].

### 4.3. Clinical Presentation of Neonatal Thyrotoxicosis and Long-Term Consequences

Newborns exhibit symptoms from birth, which can be atypical and easily missed. However, the clinical presentation may become more pronounced after 48 h, coinciding with increased activity of the 5-monodeiodinase enzyme responsible for converting T4 to T3 [21,35]. Neurological signs include excessive restlessness, hyperactivity, irritability, and sleep disorders. High thyroid hormone levels cause a hypermetabolic state, leading to fever, sweating, diarrhea, vomiting, tachypnea, arrhythmia, supraventricular tachycardia, systolic hypertension, and hypertensive encephalopathy. Physical signs can include periorbital edema, exophthalmos, lid retraction, craniosynostosis, microcephaly, and a triangular face [14]. Other possible symptoms are chylothorax, cholestasis, hepatopathy, hepatosplenomegaly, hypoglycemia, thrombocytopenia, lymphadenopathy, hip dysplasia, and prolonged acrocyanosis. Many newborns have goiter and fail to gain weight despite an excessive appetite, with reduced subcutaneous adipose tissue. Severe thyroid stimulation can lead to pulmonary hypertension, convulsions, and heart failure [14,36].

Long-term consequences of neonatal hyperthyroidism include growth retardation, hyperactivity, behavioral issues, and craniosynostosis, which may lead to cognitive impairment [26]. Elevated maternal fT4 levels during pregnancy are linked to lower child intelligence quotients, as well as reduced gray matter and cortex volume, highlighting the impact of hyperthyroidism on fetal brain development [17,26]. However, the prognosis depends on when hyperthyroidism starts and its severity [14].

### 4.4. The Role of TRAbs in Fetal/Neonatal Thyrotoxicosis

TRAbs are specific markers for GD, the leading cause of hyperthyroidism in women of childbearing age, impacting 0.4–1.0% of women before pregnancy and around 0.2% during pregnancy [37]. Its prevalence rises with maternal age, increasing from 0.5% at 30 years old to 1.3 at 40 years [38].

During pregnancy, maternal TRAb levels can indicate potential neonatal issues, as the risk of fetal/neonatal thyrotoxicosis increases with rising TRAb concentrations [39]. Therefore, close monitoring of maternal thyroid function and TRAb levels, as well as fetal ultrasounds to assess thyroid anatomy and detect signs of hyperthyroidism, is recommended in the second half of pregnancy for women with active GD or a history of GD. TRAb monitoring thresholds vary: Endocrine Society guidelines recommend levels at least two to three times above normal [40], while the American Thyroid Association suggests levels more than three times above normal [15], and a study by van Dijk et al. proposes levels 3.7 times above normal [32].

As illustrated through clinical cases [21,22,23,24,25,26,27,28,29,30], making monitoring decisions can be challenging in pregnant women without active disease or proper medical records of GD treatment. The decision becomes easier if the patient’s history indicates thyroid issues or if a clinical examination reveals signs such as goiter, neck scars, or orbitopathy [41]. Notably, TRAb production can persist for several years following ablative treatment, especially after radioiodine therapy compared to surgery. A comparison of GD treatments showed that TRAb concentrations gradually decreased with antithyroid drugs and thyroidectomy, disappearing in 70–80% of patients after 18 months [42,43]. A subsequent study of patients who underwent thyroidectomy confirmed a rapid decrease in TRAb values for most patients, particularly within the first three months following surgery [44]. In contrast, TRAb levels increased within the first months in patients who underwent radioiodine therapy, remaining above normal in 40% of patients even after five years [43].

Due to pregnancy-induced immunosuppression, TRAb concentrations typically decrease gradually in the second half of pregnancy [6]. However, case studies and limited clinical data suggest that TRAb levels may increase in certain cases [45,46], as we have also observed in our clinical cases. Currently, the mechanisms and risk factors contributing to TRAb increase during pregnancy remain unclear. One possible explanation for this increase, particularly after ablative treatment with radioiodine or surgery, is the impaired immunomodulatory function of regulatory B cells. These cells exhibit immunosuppressive effects by producing anti-inflammatory cytokines, playing a crucial role in maintaining an immunotolerant environment for the fetus during pregnancy. However, a decrease in regulatory B cells has been associated with autoimmune diseases and increased production of autoantibodies [47]. Thus, in certain pregnant women with GD, an insufficient rise in regulatory B cells could lead to a stimulated humoral response and an increase in TRAb production.

Only 10% of maternal TRAbs cross the placental barrier between the 17th and 20th gestational weeks, increasing to 50% between the 28th and 32nd weeks [13]. Consistent with this observation, our case revealed a comparable concentration of TRAbs in both the mother and the newborn at delivery. However, in neonates, TRAb concentrations decrease slowly. Given the approximate three-week half-life of TRAbs, neonatal symptoms typically improve gradually within this timeframe [17]. Consequently, clinical manifestations usually resolve within 3 to 16 weeks as maternal antibodies clear [48,49]. In our cases, TRAb concentrations decreased by approximately 50% during the first two weeks with methimazole treatment.

It is important to note that, in addition to stimulating TRAbs, which bind to and activate the TSH receptor to stimulate thyroid hormone production, blocking and neutral TRAbs also exist [10]. Blocking TRAbs inhibit TSH action, reducing thyroid hormone synthesis. Neutral TRAbs, while not affecting TSH binding, can induce thyroid cell apoptosis. Therefore, the clinical outcome depends on the presence of different proportions of high-affinity TRAbs with varied biological activities [6,13]. Stimulating and blocking TRAb activities can be distinguished using bioassays, though these are not typically employed in clinical routines [50]. Modern automated immunoassays, which are widely used and offer nearly 100% specificity and sensitivity, do not differentiate between TRAb antibody subtypes [51]. However, a recent study using a third-generation TRAb immunoassay found that third trimester maternal TRAb levels 4.6 times above the upper limit and day three neonatal TRAb levels 2.9 times above the upper limit predicted neonatal thyrotoxicosis with 100% sensitivity and 97.4% specificity [52].

### 4.5. Therapeutic Considerations in Fetal/Neonatal Thyrotoxicosis in Hypothyroid Mothers with GD

The primary treatment for TRAb-induced hyperthyroidism involves thionamide drugs, specifically methimazole, carbimazole, and propylthiouracil. They function by inhibiting thyroid hormone synthesis through interference with thyroid peroxidase, leading to reduced secretion of thyroid hormones. Propylthiouracil additionally blocks the conversion of T4 to T3 [8]. Treatment with antithyroid drugs has long been recognized for its effectiveness not only in reducing thyroid hormone levels but also in decreasing TRAb concentrations [53]. Although early studies suggested that the decrease in TRAbs is associated with the immunosuppressive effects of thionamide drugs, subsequent considerations have focused on the role of establishing euthyroidism in TRAb decline and GD remission [42,54]. The intricate effects of antithyroid drugs on hormonal and immunological mechanisms are also underscored by a recent study, which found that thymus enlargement observed in newly diagnosed GD patients during antithyroid drug treatment significantly diminishes as euthyroidism is achieved and TRAb antibody levels decrease [55]. In hyperthyroid pregnant mothers with GD, antithyroid drugs raise concerns about teratogenic defects, especially in the first trimester. If treatment is needed, propylthiouracil is preferred, with a 2–3% risk [11,15]. However, in the second half of pregnancy, methimazole is preferred over propylthiouracil due to the latter’s risk of hepatotoxicity [11]. Antithyroid drugs cross the placenta, reducing both maternal and fetal thyroid hormone production, with the goal of maintaining euthyroidism in both the mother and the fetus [6].

However, in mothers treated for hypothyroidism following ablative treatment of GD with L-T4, managing fetal thyrotoxicosis can be challenging. In this scenario, it is advantageous that antithyroid drugs cross the placenta more readily than L-T4, whose transfer is limited by placental deiodination, and exert a more pronounced effect on the fetal thyroid gland [6,56,57]. Unfortunately, research related to treatment in these challenging cases is lacking, with reports on treatment outcomes limited to rare clinical cases. In the cases described above, all three thionamide drugs were used to treat fetal thyrotoxicosis [23,25,26,28,29,30]. However, as in our cases, neonates were treated with methimazole, which a recent meta-analysis has shown to be more effective than propylthiouracil [58]. Additionally, current guidelines advise against the use of propylthiouracil in children due to the risk of hepatic failure [59]. Neonatal treatment should be adjusted based on frequent thyroid function monitoring, with dosages gradually reduced until complete withdrawal. Even TRAb-positive neonates without biochemical or clinical signs of thyroid dysfunction should undergo weekly clinical and laboratory follow-ups until TRAb levels become negative [60]. After treatment, careful follow-up is necessary, as TSH levels in newborns can remain suppressed for extended periods, potentially leading to central hypothyroidism [25].

## 5. Conclusions

Managing GD during pregnancy can be challenging both in cases of active disease and in women who have undergone ablation with radioiodine or surgery in the past, and have thyroid hormone replacement therapy properly managed. The primary concern is the activity of TRAbs that cross the placenta mainly in the second half of pregnancy, stimulating the thyroid in both the fetus and neonate. To avoid serious complications, prompt diagnosis and treatment are crucial for the mother and child. Therefore, it is essential to timely measure TRAbs in every pregnant woman with active GD or a history of GD to assess risk. Strict adherence to recommendations and close monitoring are warranted when TRAb levels are significantly elevated, including evaluation of maternal thyroid function, TRAb levels, and fetal ultrasound. Treating fetal/neonatal thyrotoxicosis poses challenges, particularly in rare cases of hypothyroid mothers with previous ablative therapy for GD. A multidisciplinary team of specialists, including endocrinologists, perinatologists, neonatologists, pediatric endocrinologists, and pediatric radiologists, should provide comprehensive follow-up care for mothers and infants to address potential risk factors before, during, and after pregnancy.

## Figures and Tables

**Table 1:** Clinical and laboratory characteristics of neonates.

	Neonate 1	Neonate 2
Mother’s characteristics	34 years	36 years
	Graves’ disease	Graves’ disease
	Graves’ orbitopathy Radioiodine therapy 12 years ago	Radioiodine therapy 14 years ago; thyroidectomy 11 years ago
Gestational age	33 weeks	35 weeks
Weight (g)/length (cm)/head circumference (cm) at birth	1550/45/28	2070/43/32
Clinical characteristics	Tachycardia (up to 220/min)	Normocardia
	Restlessness/tremor Exophthalmos Intraventricular hemorrhage	Restlessness/tremor Jaundice Mild lateral ventricular asymmetry
Laboratory tests	Day 1: TSH 0.01 mlU/L, fT4 93.3 pmol/L, fT3 22.0 pmol/L Day 6: TSH < 0.01 mlU/L, fT4 20.2 pmol/L, fT3 7.6 pmol/L, TRAb 68.7 IU/L Day 19: TSH 0.01 mlU/L, fT4 28.1 pmol/L, fT3 13.2 pmol/L, TRAb 28.7 IU/L	At birth: TSH 0.01 mlU/L, fT4 33.6 pmol/L, fT3 6.1 pmol/L, TRAb 38.9 IU/L Day 5: TSH 0.01 mlU/L fT4 49.0 pmol/L, fT3 20.7 pmol/L, TRAb 28.1 IU/L Day 10: TSH 0.01 mlU/L, fT4 27.0 pmol/L, fT3 14.5 pmol/L, TRAb 21.7 IU/L Day 17: TSH 0.01 mlU/L, fT4 15.8 pmol/L, fT3 8.3 pmol/L

TSH, thyrotropin (reference range, 0.87–6.15 mIU/L); fT4, free thyroxine (reference range, 12.1–18.6 pmol/L); fT3, free triiodothyronine (reference range, 5.1–8.0 pmol/L); TRAb, antibodies against thyrotropin receptor (reference range, below 3.1 IU/L).

## Data Availability

Data for this study are available upon reasonable request.
